# Dietary iso-α-acids prevent acetaldehyde-induced liver injury through Nrf2-mediated gene expression

**DOI:** 10.1371/journal.pone.0246327

**Published:** 2021-02-05

**Authors:** Takahito Takase, Tsudoi Toyoda, Naoyuki Kobayashi, Takashi Inoue, Tomoko Ishijima, Keiko Abe, Hiroshi Kinoshita, Youichi Tsuchiya, Shinji Okada

**Affiliations:** 1 Research and Development Division, SAPPORO HOLDINGS LTD., Yaizu, Shizioka, Japan; 2 Fundamental Laboratory, POKKA SAPPORO FOOD & BEVERAGE LTD., Yokohama, Kanagawa, Japan; 3 Graduate School of Agricultural and Life Sciences, The University of Tokyo, Bunkyo-ku, Tokyo, Japan; 4 Department of Forensic Medicine, Faculty of Medicine, Kagawa University, Miki, Kita, Kagawa, Japan; National Institutes of Health, UNITED STATES

## Abstract

Acetaldehyde is the major toxic metabolite of alcohol (ethanol) and enhances fibrosis of the liver through hepatic stellate cells. Additionally, alcohol administration causes the accumulation of reactive oxygen species (ROS), which induce hepatocyte injury-mediated lipid peroxidation. Iso-α-acids, called isohumulones, are bitter acids in beer. The purpose of this study was to investigate the protective effects of iso-α-acids against alcoholic liver injury in hepatocytes in mice. C57BL/6N mice were fed diets containing isomerized hop extract, which mainly consists of iso-α-acids. After 7 days of feeding, acetaldehyde was administered by a single intraperitoneal injection. The acetaldehyde-induced increases in serum aspartate aminotransferase (AST) and alanine aminotransferase (ALT) levels were suppressed by iso-α-acids intake. Hepatic gene expression analyses showed the upregulation of detoxifying enzyme genes, glutathione-S-transferase (GST) and aldehyde dehydrogenase (ALDH). In vitro, iso-α-acids upregulated the enzymatic activities of GST and ALDH and induced the nuclear translocation of nuclear factor-erythroid-2-related factor 2 (Nfe2l2; Nrf2), a master regulator of antioxidant and detoxifying systems. These results suggest that iso-α-acid intake prevents acetaldehyde-induced liver injury by reducing oxidative stress via Nrf2-mediated gene expression.

## Introduction

Excessive consumption of alcoholic beverages is a leading cause of liver disease, including cirrhosis, liver cancer, and acute and chronic liver failure, worldwide [1]. The metabolism of ethanol generates reactive oxygen species (ROS), which play a role in the deterioration of alcoholic liver disease (ALD) [2]. There was a positive correlation between individual alcohol consumption and the ratio of ALD patients to all liver disease patients, and both are increasing in Asia [3]. Globally, in 2010, alcohol-attributable liver cirrhosis was responsible for 493,300 deaths. And alcohol-attributable liver cirrhosis was responsible for 225,900 deaths in Asia [3].

Ingested alcohol (ethanol) is absorbed mainly in the small intestine and, passing through the liver, is distributed throughout the entire organism. Ethanol is oxidized to acetaldehyde by alcohol dehydrogenase (ADH), and acetaldehyde is further oxidized to acetic acid by aldehyde dehydrogenase (ALDH) [4]. There are 2 major ALDH isoforms, cytosolic ALDH (ALDH1) and mitochondrial ALDH (ALDH2), and acetaldehyde oxidation is almost entirely dependent on ALDH2 in the human liver. East Asian populations such as the Japanese, Chinese and Korean populations harbor an allele (ALDH2*2) of the ALDH2 gene that contains a single-nucleotide polymorphism and results in the synthesis of an inactive ALDH2 enzyme [5]. After ethanol consumption, ALDH2 deficiency causes increased accumulation of acetaldehyde in the blood and saliva [6].

Acetaldehyde binds to glutathione (GSH), reduces antioxidant activity and promotes the production of ROS [7]. Chronic alcohol consumption enhances cytochrome P450E1 activity and produces hyperoxide [8]. Hyperoxide is converted into hydrogen peroxide by superoxide dismutase (SOD), and it is a source of strong oxidative stress. The ROS produced can cause lipid peroxidation of cellular membranes and protein and DNA oxidation, which result in alcoholic liver disease [9]. Aspartate aminotransferase (AST) and alanine aminotransferase (ALT) are present in hepatocytes, and their levels in serum are altered by liver damage. Heavy drinking of ethanol induces increased serum ethanol concentrations and levels of liver function markers such as AST and ALT [10].

Nuclear erythroid 2-related factor 2 (Nfe2l2; Nrf2) is a transcription factor activated by oxidative stress, such as ROS and electrophiles, and regulates antioxidant gene expression [11]. A previous study showed that the enhanced expression of Nrf2 in mice prevents oxidative stress and ameliorates ALD through the modulation of antioxidant defense-associated genes [12]. Studies in Nrf2-knockout mice showed that Nrf2 deficiency enhanced alcohol-induced liver injury compared with Nrf-2 levels in control mice [13]. Thus, Nrf2-mediated antioxidant effects might be associated with protective effects against ALD.

Hop cones, the female flowers of *Humulus lupulus* L., are used as a preservative and flavoring in beer. Iso-α-acids, called isohumulones, are bitter acids in beer and result from the oxidation of hop-derived α-acids [14]. Iso-α-acids contribute to the preservation of beer by antibacterial activity [15]. Iso-α-acids have antioxidative activity and reduce metabolic inflammation and insulin resistance. Iso-α-acids improved blood vessel endothelium via reduction of the reactive oxygen species (ROS) in endothelial cell and human [16]. Intake of iso-α-acids reduced plasma glucose and triglyceride levels in high fat diet-fed mice [17]. And dietary supplementation with iso-α-acids was found to reduce the production of the proinflammatory prostaglandin E2 in the rat [18].

It was difficult to detect acetaldehyde in blood of ethanol administered mice. Blood acetaldehyde was detected in acetaldehyde intraperitoneal injected mice, but it was not detected in ethanol administered mice [19]. In this study, to investigate the effects of iso-α-acids on acetaldehyde metabolism and acetaldehyde-induced liver injury, we examined the influence of iso-α-acids consumption in acetaldehyde-treated mice.

## Materials and methods

### Materials

All reagents used in this study were commercial-grade specific reagents.

### Preparation of iso-α-acids

Iso-α-acids were prepared from hop extract (Hop Extract No. 102536; San-Ei Gen F.F.I., Inc., Japan). Hop extract was suspended in 1 N HCl for partitioning with hexane. We collected the hexane layer and prepared the extract, which included iso-α-acids in high density (isohumulones content of more than 70% by HPLC analysis), by distilling off the hexane in an evaporator, and we used the extract for experiments [14].

### Animal experiment

Eight-week-old male C57BL/6NCrSlc mice were purchased from Japan SLC (Shizuoka, Japan) and were housed under controlled temperature (24 ± 1°C), humidity (50–70%), and light (12 h light-dark cycle) conditions. The protocols for the animal experiments were approved by the Animal Use Committee of Sapporo Holdings Ltd., Research and Development Division (permission number: 2018–004).

The eight-week-old male C57BL/6NCrSlc mice were fed the AIN-93G diet (Oriental Yeast, Tokyo, Japan) for a week and then were divided into two groups (*n* = 15 per group) based on similar average body weight. The control diet group was fed the AIN-93G diet, and the iso-α-acid diet group was fed the AIN-93G diet containing 0.5% (w/w) iso-α-acids. Mice in the control group were pair-fed the control diet in the amount of the ad libitum intake of the iso-α-acids group for a week. After a week feeding period, animals were treated with an intraperitoneal (i.p.) injection of acetaldehyde (200 mg/kg) [19]. For serum AST and ALT assay and liver tissue collection, tail vein blood samples were collected at 0, 1, 3, and 5 h after the acetaldehyde injection (200 mg/kg, i.p.) and liver samples were collected after blood sampling (5 h time point) under deep anesthesia (*n* = 10). For blood acetaldehyde concentration measurement, orbital sinus blood samples were collected at 5, 10, and 15 min intervals after the acetaldehyde injections (*n* = 5).

### Serum enzyme assays

Serum was prepared from tail vein blood samples. Serum AST and ALT activity was measured using a transaminase CII test kit (Wako, Osaka, Japan).

### Determination of Aldehyde Dehydrogenase (ALDH) activity

The ALDH activity of mouse liver tissues and murine hepatoma cells was determined by measuring the 6-methoxy-2-naphthoic acid that produced 6-methoxy-2-naphthaldehyde by the oxidation of the aldehyde group using HPLC with fluorescence detection [20]. We used the Agilent 1100 HPLC system (Agilent Technologies Japan, Tokyo, Japan) equipped with a Symmetry C18 HPLC column (2.1 × 50 mm, 3.5 μm: Nihon Waters, Tokyo, Japan). The separation of compounds was carried out by gradient elution. Solvent A was 1.0% formic acid, and solvent B was acetonitrile containing 1.0% formic acid. The gradient program was as follows: 0 min, 36% B; 0–4.5 min, linear gradient to 54% B. The flow was 0.5 mL/min, the column temperature was 40°C, and naphthoic acid was detected by fluorescence (Ex. 310 nm/Em. 360 nm).

### Determination of Glutathione S-Transferase (GST) activity

The GST activity of mouse liver tissues and Hepa1c1c7 cells was determined by the method of Habig WH *et al*. [21]. The assay mixture (180 μL) contained 100 mM potassium phosphate (pH6.5), 1.0 mM GSH and 10 μL liver or cell fraction. The assay was started by addition of 20 μL of 10 mM 1-chloro-2,4-dinitrobenzene, bringing the total volume to 200 μL. The initial velocity of 2,4-dinitrophenyl-S-glutathione generation was measured its absorbance at 340 nm.

### Determination of lipid hydroperoxides

Levels of lipid hydroperoxide, such as malondialdehyde (MDA), in mouse liver tissues were assessed by the thiobarbituric acid reactive substances (TBARS) assay [22]. The assay mixture (150 μL) contained 0.375% thiobarbituric acid, 15% trichloroacetic acid, 0.4% butylated hydroxytoluene, 0.25N HCl and 50 μL liver fraction. The assay mixture was boiled for 15 minutes. The reaction of MDA with thiobarbituric acid was detected by spectrometry (532 nm).

### Determination of blood acetaldehyde levels

Collected orbital sinus blood samples were immediately added to 500 μL 0.001% (w/w) t-butanol as an internal standard consisting of 0.6 N perchloric acid, vortexed and then centrifuged at 1,000×g for 3 min. Finally, 450 μL of the supernatant was transferred to 20 mL gas chromatography vials and used to determine blood acetaldehyde levels by gas chromatograph (GC). A GC equipped with a flame ionization detector (GC-2014, Shimazu, Japan) combined with a head space auto sampler (TurboMatrix 40, PerkinElmer) was used throughout the study. The chromatographic conditions, in short, were as follows: the column, injector and detector temperatures were 90, 110, and 200°C, respectively. The separation column was a Supelcowax wide bore capillary (60 m length, 0.53 mm i.d., 2 μm film thickness, Supelco, PA, USA). Nitrogen was used as the carrier gas at 50 kPa [19].

### DNA microarray experiment

Total RNA was isolated from each liver sample using TRIzol reagent (Thermo Fisher Scientific, Waltham, MA, USA) and was subsequently purified using an RNeasy Mini Kit (Qiagen, Hilden, Germany) and RNase-Free DNase Set (Qiagen), according to the manufacturer’s protocol. Total RNA quality and quantity were assessed by agarose gel electrophoresis and spectrophotometry.

DNA microarray analysis was performed on liver samples from a total of ten mice (five mice each from the iso-α-acids and control diet groups) by choosing representative individuals from each group on the basis of their serum enzyme values.

For each sample, biotinylated single-stranded cDNA was synthesized from 100 ng of total RNA using a GeneChip WT PLUS reagent kit (Thermo Fisher Scientific). The cDNAs were subsequently hybridized to a Clariom S Mouse Array (Thermo Fisher Scientific). The arrays were washed and labeled with streptavidin–phycoerythrin using a GeneChip Hybridization, Wash and Stain Kit and the Fluidics Station 450 system (Thermo Fisher Scientific). Fluorescence was detected using a GeneChip Scanner 3000 7G (Thermo Fisher Scientific).

Affymetrix GeneChip Command Console software was used to reduce the array images to the intensity of each probe (CEL files). The CEL files were quantified using the Factor Analysis for Robust Microarray Summarization algorithm (quantile normalization, qFARMS) [23] with the statistical packages R [24] and Bioconductor [25]. Probe sets found to be differentially expressed between the iso-α-acids and control diet groups were identified according to the rank products method [26] using R. All microarray data were deposited in the National Center for Biotechnology Information Gene Expression Omnibus (http://www.ncbi.nlm.nih.gov/geo/; GEO Series accession number GSE140387).

Probe sets with a false-discovery rate (FDR) < 0.05 were considered to reflect the intake of the iso-α-acids. Gene-annotation enrichment analysis was then performed using the web tool Database for Annotation, Visualization, and Integrated Discovery (DAVID; http://david.abcc.ncifcrf.gov/) with Gene Ontology (GO). Benjamini-Hochberg FDR corrections were used to correct the results. GO terms with FDR-corrected *p*-values of < 0.01 were regarded as significantly enriched. Subsequently, Ingenuity Pathway Analysis (IPA, Qiagen) was used to identify activated/inactivated canonical pathways and upstream regulators by iso-α-acid intake. IPA calculated *p*-values using Fisher’s exact test. In addition, IPA also calculated activation z-scores. Canonical pathways and upstream regulators with a *p*-value < 0.05 were regarded as statistically significant. The pathways and upstream regulators with z-scores > 2 were regarded as significantly activated, and those with z-scores < -2 were regarded as significantly inactivated.

### Quantitative RT-PCR analysis

Total RNA isolated from liver samples was used. The cDNA synthesis of total RNA was carried out using a ReverTra Ace qPCR RT kit (TOYOBO, Osaka, Japan) according to the manufacturer’s instructions. To measure the mRNA amount in liver samples, RT-PCR was conducted with SYBR green dye using the LightCycler 480 SYBR system (Roche Applied Science, Mannheim, Germany). The oligonucleotide primers used for amplification were obtained from commercial products (*Adh1*, *Aldh1*, *Aldh2*, *Gsta2*, *Gsta4*, *Gstm1*, *Gstt2*, *Sod1* and *Actb* (β-actin) (Takara Bio, Shiga, Japan)). The expression of each gene was normalized to that of β-actin mRNA.

### Cell culture and treatment

Hepa1c1c7 (ECACC 95090613) murine hepatoma cells were cultured with alpha modified Eagle minimum essential medium supplemented with 10% fetal bovine serum, 100 units/mL penicillin, and 100 μg/mL streptomycin in an atmosphere of 5% CO^2^ at 37°C.

Cells were seeded on 6-well plates at a density of 2.5 × 10^5^ cells/well for each experiment and allowed to grow for 24 h. Cells were then treated with either vehicle (acetonitrile, 0.1%) or various concentrations (5, 25 or 100 ppm) of iso-α-acids for 48 h. The experimental conditions were in accord with a previous report [27].

### Western blotting

To examine the nuclear translocation of the transcription factor Nrf2, nuclear proteins were isolated from treated Hepa1c1c7 cells by using a LysoPure Nuclear and Cytoplasmic Extractor Kit (Wako).

Nuclear proteins were separated by 10% sodium dodecyl sulfate-polyacrylamide gel electrophoresis (SDS-PAGE). Proteins were transferred onto polyvinylidene difluoride membranes by using the Trans-Blot Turbo Transfer System (Bio-Rad Hercules, CA, USA), followed by blocking of nonspecific binding with 5% skim milk in Tris-buffered saline. Membranes were incubated with antibodies against Nrf2 (1:1,000; Cell Signaling Technology Japan, Tokyo, Japan) or β-actin (1:10,000; Cell Signaling Technology Japan) at 4°C overnight. After washing with Tris-buffered saline containing 0.05% Tween 20, membranes were incubated with peroxidase-labeled secondary antibody (1:5,000 anti-rabbit) for 1 h, and then immune complexes were visualized using ECL Prime Western Blotting Detection Reagent (GE Healthcare Japan, Tokyo, Japan) and analyzed by the ChemiDoc XRS system (Bio-Rad).

### Statistical analysis

All values are expressed as the mean ± standard error (S.E.M) of the mean. Statistical analysis was performed using Student’s t test and Dunnett’s test where appropriate by using JMP 13 (SAS Institute Japan, Tokyo, Japan). Differences were considered significant at *p* < 0.05.

## Results

### The preventive effects of iso-α-acid intake on acetaldehyde-induced liver injury

**Fig 1:**
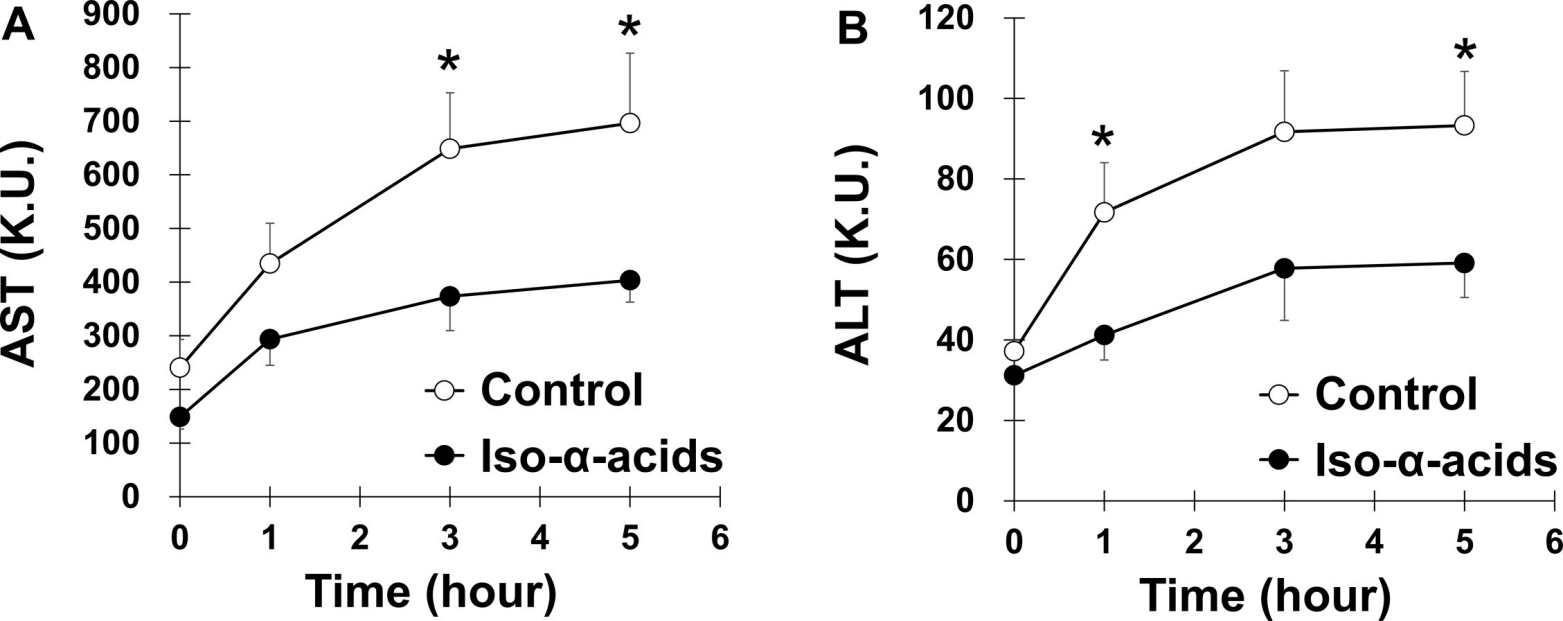
The effect of iso-α-acids on acetaldehyde-induced liver injury. Mice fed the control diet or the iso-α-acids diet for a week were treated with acetaldehyde (200 mg/kg, i.p.). Time course of serum AST (A) and ALT (B) levels after acetaldehyde treatment are shown. Data are represented as the mean ± S.E.M (*n* = 10). *p < 0.05 (vs. the control group).

To evaluate the effects of iso-α-acids administration on acetaldehyde-induced liver injury, mice were fed iso-α-acids or a control diet for one week before administration of a single i.p. injection of acetaldehyde (200 mg/kg). Their body weights and liver weights were not different between the two groups (S1 Table). We measured acetaldehyde i.p. injection-induced changes in serum AST and ALT levels of the mice. The administration of acetaldehyde lead to increased serum AST and ALT levels. The elevations in both AST and ALT levels were significantly lower in the iso-α-acid diet group than in the control diet group (Fig 1). This result suggested that the intake of iso-α-acids provided preventive effects in mouse liver against acetaldehyde-induced injury.

### Intake of iso-α-acids upregulated the gene of detoxification, antioxidation and ethanol degradation

To characterize the mechanism underlying the preventive effects of iso-α-acids on acetaldehyde-induced liver disease, the global gene expression in mouse liver was analyzed by a DNA microarray technique. Principal component analysis of DNA microarray data revealed that mice fed the iso-α-acids diet and control diet formed clusters that were distinct from each other (S1 Fig). This result showed that the iso-α-acids diet significantly influenced the gene expression profile in mouse liver. We extracted differentially expressed genes (DEGs) statistically using the rank products method and identified 851 upregulated probe sets and 714 downregulated probe sets in the iso-α-acid diet group compared to the control diet group, with an FDR of < 0.05.

**Fig 2:**
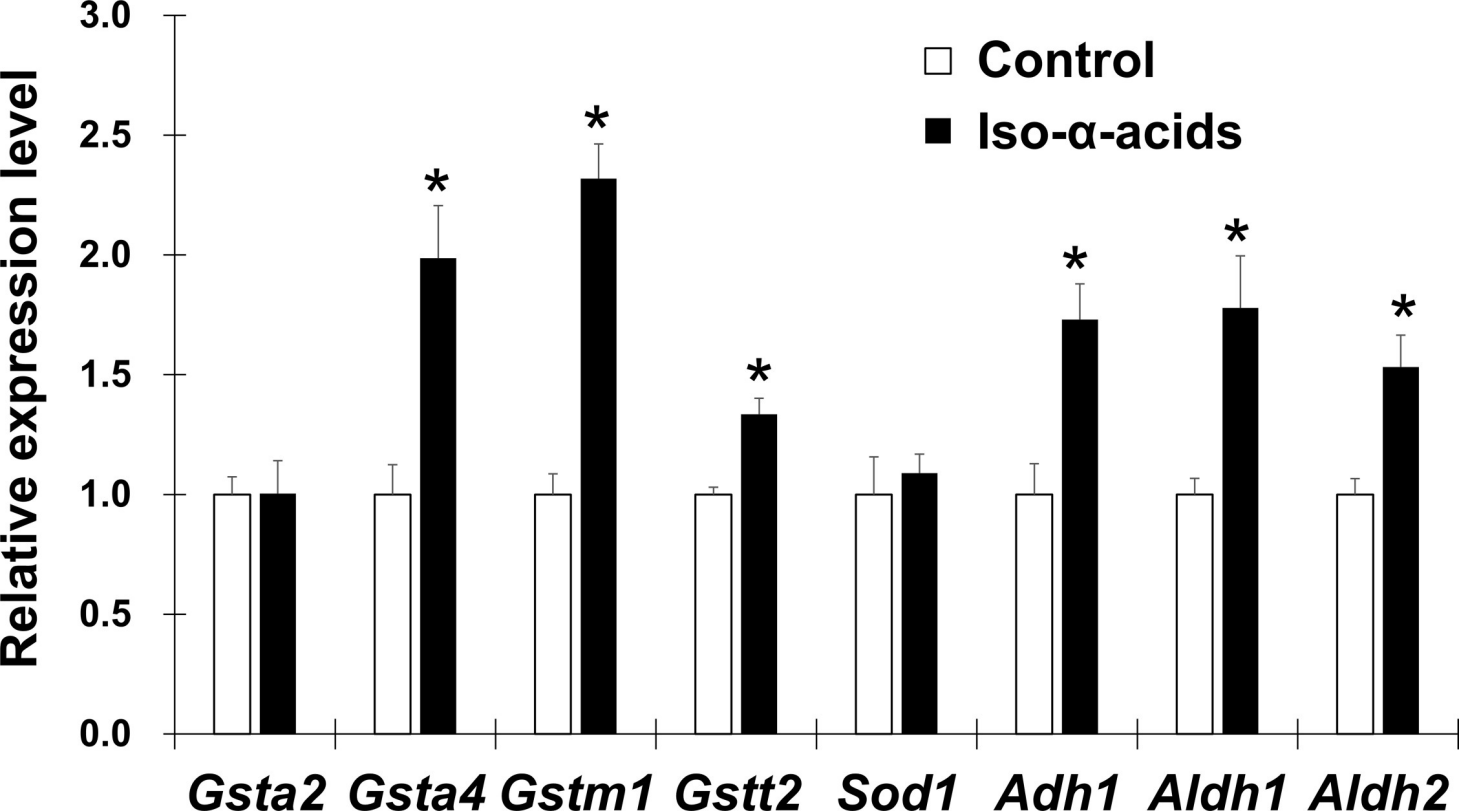
The effect of iso-α-acids on mRNA expression of genes related to the glutathione metabolic process, oxidative stress response and aldehyde metabolism in the liver. Mice fed the control diet or the iso-α-acids diet for a week were treated with acetaldehyde (200 mg/kg, i.p.) for 5 hours. mRNA levels were examined by quantitative RT-PCR and normalized to β-actin mRNA expression. The relative expression levels of genes were calculated as the ratio of levels in the iso-α-acids group relative to those in the control group. Data are shown as the mean ± S.E.M (*n* = 5). *p < 0.05 (vs. the control group).

DEGs were classified into functional categories according to Gene Ontology (GO) biological process terms using a modified Fisher’s exact test (FDR < 0.01). The significantly enriched GO terms found in the upregulated and downregulated genes were applied to QuickGO to map the terms hierarchically (S2 and S3 Figs). To select DEGs related to liver injury, we focused on the upregulated genes including GO terms related to “glutathione metabolic process (GO:0006749)” (Table 1). The expression of genes in the GST family was upregulated by the intake of iso-α-acids. The GST family are Phase II drug-metabolizing enzymes and have the function of detoxification and antioxidation. Next, DEGs were imported into the IPA software to identify biological networks and pathways (Table 2). This pathway analysis also showed the expression changes in genes in detoxification and antioxidation pathways such as “Nrf2-Mediated Oxidative Stress Response”, “Glutathione-Mediated Detoxification” and “Glutathione Redox Reactions” pathways. These pathways were predicted to be activated. Furthermore, the analysis predicted the activation of ethanol degradation pathways. DNA microarray analysis and IPA revealed that the expression of genes related to detoxification, antioxidation and ethanol degradation were upregulated in the liver by iso-α-acid intake. Therefore, we analyzed the expression of these genes in the liver by quantitative RT-PCR analysis (Fig 2). The expression levels of *Gsta4*, *Gstm1*, *Gstt2*, *Adh1*, *Aldh1* and *Aldh2* were significantly increased in the iso-α-acid diet group compared to the control diet group. However, the expression levels of *Gsta2* and Sod1 were not increased. These results indicated that the intake of iso-α-acids upregulated the expression levels of genes related to detoxification, antioxidation and ethanol-acetaldehyde degradation in mouse liver.

**Table 1:** Upregulated genes related to the glutathione metabolic process that are altered in the liver following the consumption of an iso-α-acids diet by a DNA microarray analysis.

Gene name	Gene symbol	FDR
***5-Oxoprolinase***	*Oplah*	4.9.E-04
***Aldehyde dehydrogenase family 5*, *subfamily A1***	*Aldh5a1*	1.5.E-03
***Cystathionase***	*Cth*	1.5.E-02
***Ethylmalonic encephalopathy 1***	*Ethe1*	7.4.E-04
***Glutamate-cysteine ligase*, *catalytic subunit***	*Gclc*	3.9.E-04
***Glutamate-cysteine ligase*, *modifier subunit***	*Gclm*	3.0.E-02
***Glutathione S-transferase omega 1***	*Gsto1*	2.8.E-03
***Glutathione S-transferase*, *alpha 2***	*Gsta2*	1.1.E-09
***Glutathione S-transferase*, *alpha 3***	*Gsta3*	9.2.E-03
***Glutathione S-transferase*, *alpha 4***	*Gsta4*	2.8.E-04
***Glutathione S-transferase*, *mu 1***	*Gstm1*	1.2.E-05
***Glutathione S-transferase*, *mu 2***	*Gstm2*	1.8.E-07
***Glutathione S-transferase*, *mu 4***	*Gstm4*	2.4.E-08
***Glutathione S-transferase*, *theta 2***	*Gstt2*	9.8.E-09
***Glutathione peroxidase 4***	*Gpx4*	5.1.E-04
***Glutathione reductase***	*Gsr*	1.5.E-04
***Isocitrate dehydrogenase 1***	*Idh1*	1.2.E-02
***Superoxide dismutase 1***	*Sod1*	1.1.E-02
***Superoxide dismutase 2***	*Sod2*	7.4.E-03

**Table 2:** Significantly activated/inactivated canonical pathways in the DEGs induced by iso-α-acids intake.

IPA canonical pathways	*p*-value	Z-score
**EIF2 Signaling**	4.0E-24	activated
**LPS/IL-1-Mediated Inhibition of RXR Function**	1.3E-22	inactivated
**Superpathway of Cholesterol Biosynthesis**	1.3E-17	activated
**Fatty Acid β-oxidation I**	1.3E-12	activated
**Nrf2-Mediated Oxidative Stress Response**	4.0E-12	activated
**Cholesterol Biosynthesis I**	5.8E-10	activated
**Cholesterol Biosynthesis II**	5.8E-10	activated
**Cholesterol Biosynthesis III**	5.8E-10	activated
**Glutathione-Mediated Detoxification**	7.6E-10	activated
**Serotonin Degradation**	7.1E-09	activated
**Mevalonate Pathway I**	2.2E-08	activated
**Ethanol Degradation II**	2.8E-08	activated
**Triacylglycerol Degradation**	3.7E-08	activated
**Superpathway of Geranylgeranyldiphosphate Biosynthesis I**	5.1E-08	activated
**Valine Degradation I**	2.4E-07	activated
**Noradrenaline and Adrenaline Degradation**	6.2E-07	activated
**Ketogenesis**	1.9E-06	activated
**Isoleucine Degradation I**	3.1E-06	activated
**Glutathione Redox Reactions I**	9.5E-06	activated
**Glutaryl-CoA Degradation**	1.1E-05	activated
**Triacylglycerol Biosynthesis**	1.9E-05	activated
**Stearate Biosynthesis I**	1.9E-05	activated
**Oxidative Ethanol Degradation III**	5.4E-05	activated
**γ-Linolenate Biosynthesis II**	7.2E-05	activated
**Mitochondrial L-Carnitine Shuttle Pathway**	1.2E-04	activated
**Tryptophan Degradation X**	1.3E-04	activated
**Zymosterol Biosynthesis**	1.9E-04	activated
**TCA Cycle II**	2.6E-04	activated
**Ethanol Degradation IV**	2.6E-04	activated
**Ketolysis**	2.1E-03	activated
**Phosphatidylglycerol Biosynthesis II**	3.5E-03	activated
**Putrescine Degradation III**	3.7E-03	activated
**Oleate Biosynthesis II**	8.3E-03	activated
**Histamine Degradation**	8.7E-03	activated
**CDP-Diacylglycerol Biosynthesis I**	9.8E-03	activated
**Fatty Acid Activation**	1.2E-02	activated
**Acyl-CoA Hydrolysis**	1.2E-02	activated

DEGs obtained by a DNA microarray analysis were enriched (p-value < 0.05) and predicted to be activated/inactivated (|activation Z-score| > 2) in 37 IPA canonical pathways.

### Intake of iso-α-acids accelerated acetaldehyde metabolism

**Fig 3:**
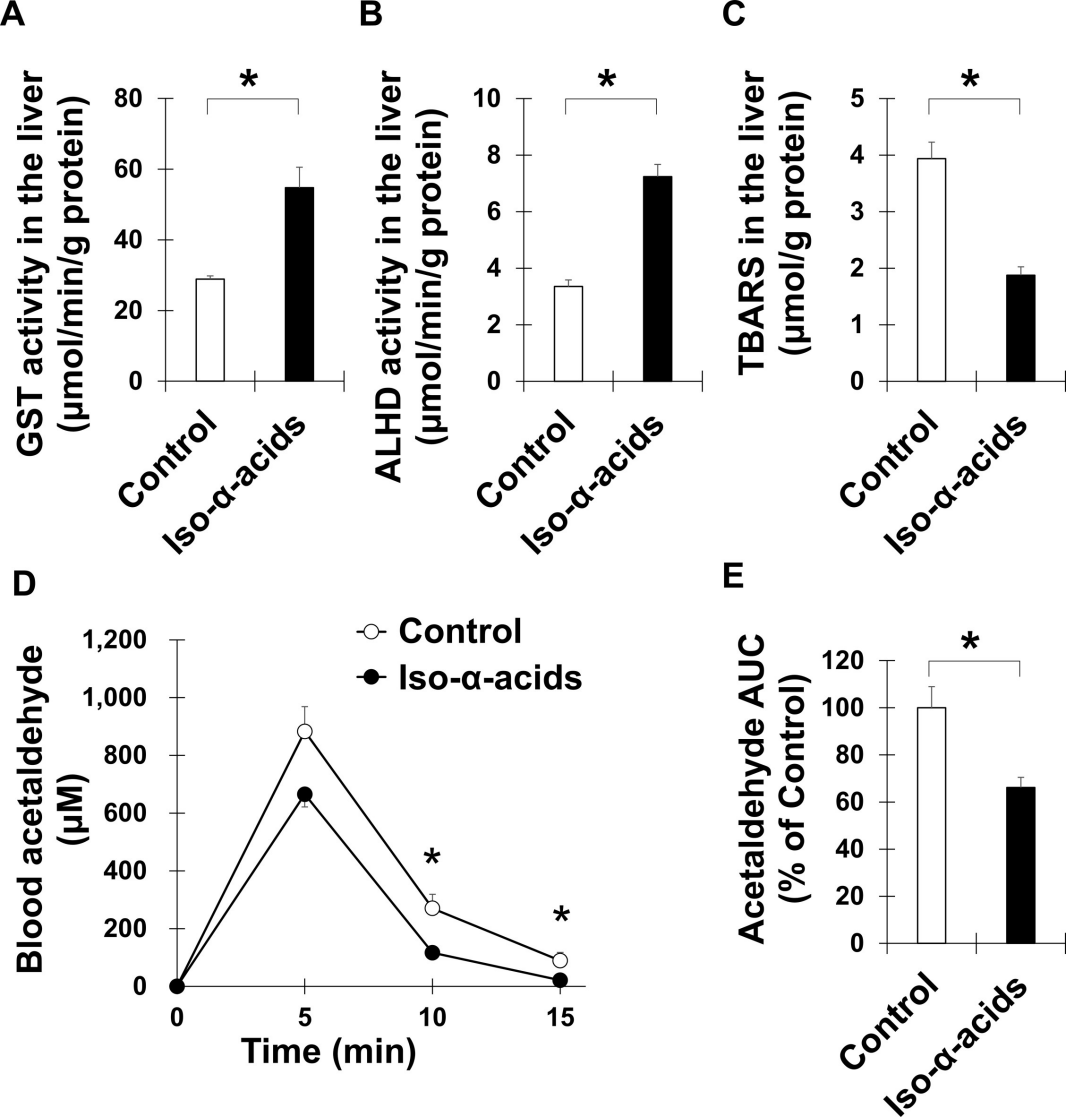
The effect of iso-α-acids on hepatic GST activity, ALDH activity, oxidative stress and blood acetaldehyde metabolism. Mice fed the control diet or the iso-α-acids diet for a week were treated with acetaldehyde (200 mg/kg, i.p.). The GST activity (A), ALDH activity (B) and TBARS (C) levels of the liver tissues 5 hours after the treatment were examined (*n* = 10). (D) Time course of the acetaldehyde concentrations of the blood samples after acetaldehyde treatment were examined (*n* = 5). (E) Areas under the curve were calculated by trapezoidal rule from 0 to 15 min. Data are represented as the mean ± S.E.M. *p < 0.05 (vs. the control group).

To evaluate the effects of iso-α-acids intake on detoxification, antioxidation and acetaldehyde metabolism, the liver tissue samples were collected 5 hours after acetaldehyde treatment and prepared. GST and ALDH activities were significantly increased in the iso-α-acids diet group (Fig 3A and 3B). These results indicated that the intake of iso-α-acids provided strong antioxidative, detoxication and acetaldehyde degradation effects to mouse liver at the enzymatic activity level.

Next, the level of TBARS, a biomarker of oxidative stress, in the liver was measured. The level of TBARS was reduced by iso-α-acid ingestion (Fig 3C). The time course of blood acetaldehyde concentrations was measured in the control diet and the iso-α-acids diet mice. The iso-α-acid diet mice exhibited accelerated acetaldehyde metabolism compared to the control diet mice (Fig 3D and 3E). These results strongly suggested that the enhanced enzymatic activities in the liver facilitated a reduction in reactive oxygen species and the degradation of acetaldehyde in mice fed the iso-α-acids diet.

### Nrf2 accumulation in iso-α-acids treated murine hepatocyte cells

Finally, we focused on the transcription factor Nrf2/Nfe2l2 as an upstream regulator of the expression of genes related to detoxification, antioxidation and ethanol-acetaldehyde degradation in the livers of mice fed the iso-α-acids diet. IPA canonical pathway analysis of DEGs in a DNA microarray analysis predicted the activation of the “Nrf2-Mediated Oxidative Stress Response” pathway as mentioned above. IPA upstream analysis predicted the activation of Nrf2 as an upstream regulator of the DEGs (activation Z-score: 5.506). These results strongly suggested the contribution of Nrf2 to the hepatic gene expression changes induced by iso-α-acids intake.

**Fig 4:**
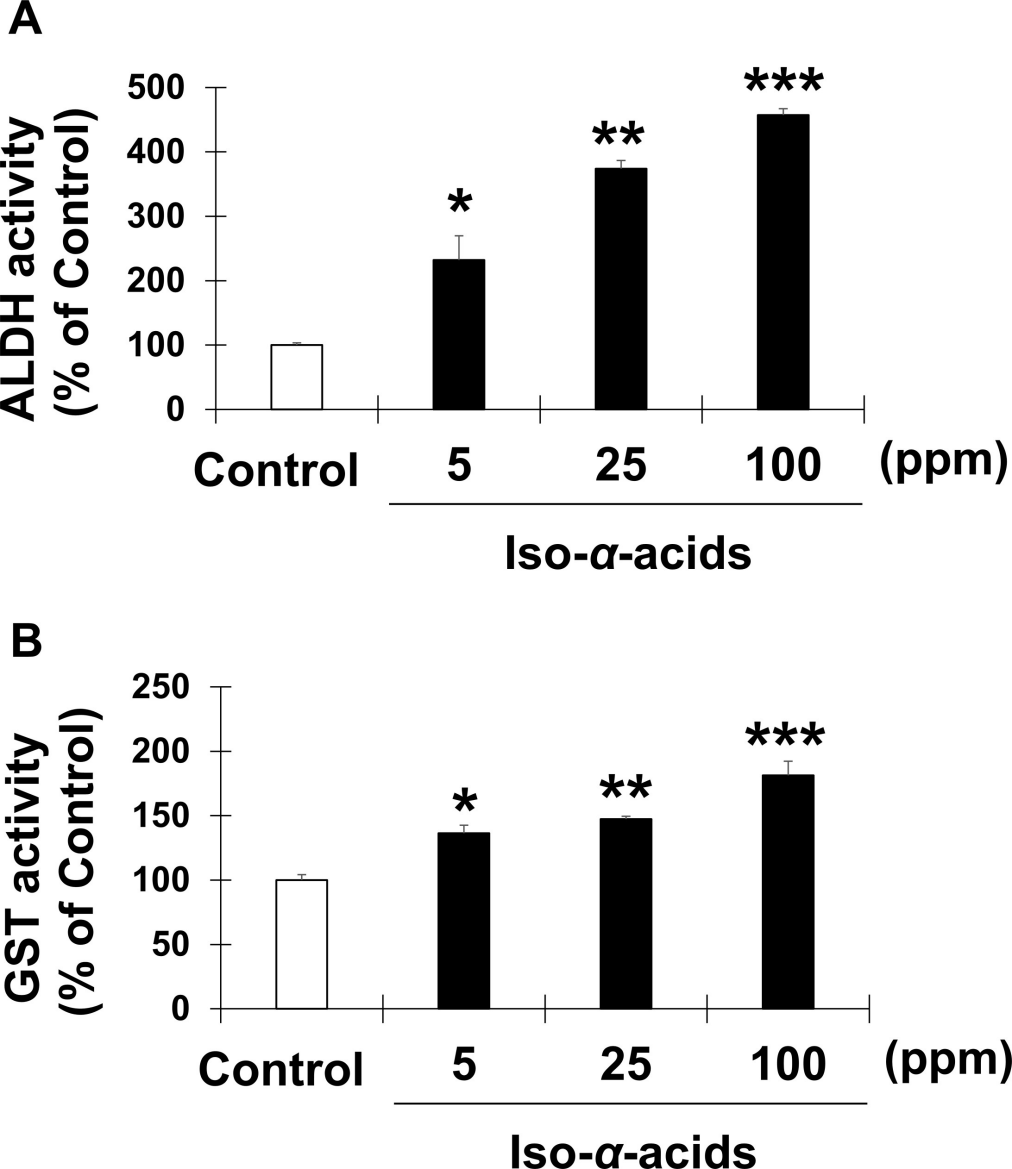
The effect of iso-α-acid treatment on GST and ALDH activity in murine hepatoma cells. Hepa1c1c7 cells were treated with iso-α-acids (5, 25 or 100 ppm) for 48 hours. The GST activity (A) and ALDH activity (B) levels of the cell lysates were examined. Data are shown as the mean ± S.E.M (*n* = 3). *p < 0.05, **p < 0.01, ***p < 0.001 (vs. the control group).

**Fig 5:**
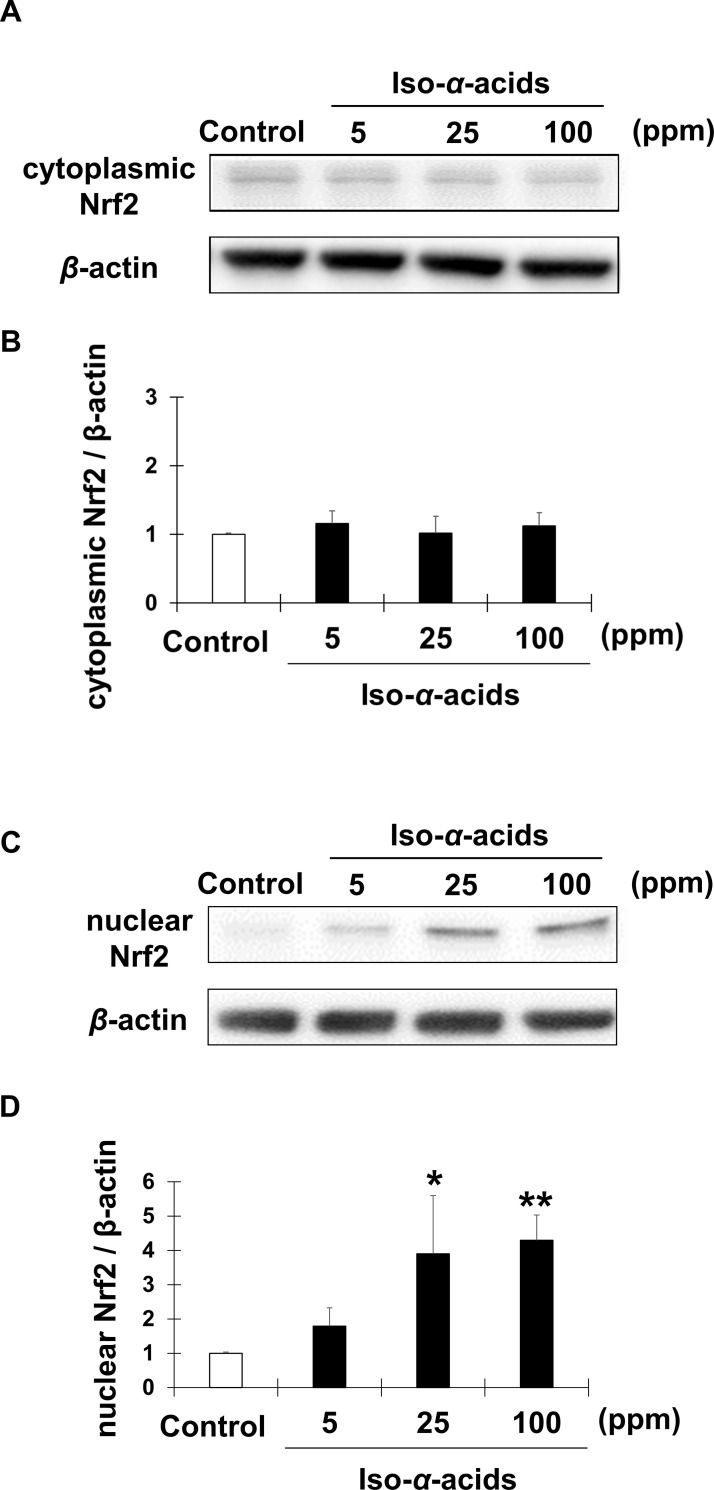
The effect of iso-α-acid treatment on nuclear translocation of Nrf2 in murine hepatoma cells. Hepa1c1c7 cells were treated with iso-α-acids (5, 25 or 100 ppm) for 48 hours. Nrf2 protein levels in the cytoplasmic fraction (A) and the nuclear fraction (C) of the cells were examined by Western blotting. Representative images of 3 samples are shown. (B) The graph shows the densitometric quantification of Western blot bands in the cytoplasmic Nrf2. (D) The graph shows the densitometric quantification of Western blot bands in the nuclear Nrf2. Data are shown as the mean ± S.E.M (n = 3). *p < 0.05, **p < 0.01, ***p < 0.001 (vs. the control group).

The accumulation of Nrf2 in the nucleus leads to the expression of antioxidant genes. However, the gene expression levels of Nrf2 and its specific repressor Kelch-like ECH-associated protein 1 (Keap1) were not changed in the liver by iso-α-acids intake in a DNA microarray analysis. Therefore, we examined the nuclear translocation of Nrf2 protein by using a murine hepatocyte cell line with iso-α-acids administration. First, we measured the GST and ALDH activities of the iso-α-acid-treated Hep1c1c7 cell lysates (Fig 4A and 4B). Overall GST and ALDH activities were enhanced by iso-α-acids treatment in vitro as well as in the liver. In vitro analysis revealed that their activities were dose dependent. Even 5 ppm of iso-α-acids administration significantly enhanced both GST and ALDH activities. Next, Western blot analysis with the nuclear fraction of the iso-α-acids treated cells was performed. The results showed that iso-α-acids induced Nrf2 accumulation in the nuclear fraction of Hepa1c1c7 cells in a dose-dependent manner (Fig 5C and 5D). These results indicated that iso-α-acids upregulated the GST and ALDH activities and facilitated the nuclear translocation of Nrf2 in hepatocytes.

## Discussion

In this study, we found that iso-α-acids intake suppressed the increase in serum AST and ALT induced with acetaldehyde. Furthermore, we revealed that iso-α-acids increased GST and ALDH activities through the Nrf2-mediated upregulation of gene expression. As a result, we demonstrated that iso-α-acid intake contributed to the hepatoprotective effect.

A previous study showed that iso-α-acids-rich extracts attenuated alcohol-induced hepatic steatosis but did not affect hepatic markers, such as AST and ALT [28]. However, we showed that AST and ALT levels were significantly decreased in the iso-α-acids diet group compared with the control diet group (Fig 1). We speculated that the differences were caused by the exposure to ethanol rather than acetaldehyde. The authors in the previous study administered one oral bolus of ethanol, while we administered acetaldehyde i.p. to induce liver injury in mice. Because the ALDH activity in mouse liver is very high, small traces of acetaldehyde were seen in mouse blood after ethanol injection [19]. Therefore, liver injury in mice might have been insufficient in the previous study. Acetaldehyde injection induced a higher accumulation of acetaldehyde in the blood than ethanol injection [19]. In fact, the AST and ALT levels after acetaldehyde administration in this study were higher than those after ethanol administration in the previous study.

GST catalyzes the reaction of reduced glutathione with xenobiotics. Additionally, the GST molecular species exert an antioxidative effect by working as hydrogen peroxide scavengers [29]. GST subsets are categorized into seven classes (alpha, mu, pi, theta, sigma, zeta and omega) [30]. Gsta4, of the alpha class, is a key enzyme for the removal of 4-hydroxy nonenal (4-HNE), produced by lipid peroxide [31, 32]. Gstt2, of the theta class, has glutathione peroxidase activity and reduces lipid peroxide to alcohol [33]. A previous study reported that GST activated by quercetin can protect against acute alcohol-induced liver injury in mice [34]. Our results showed that iso-α-acids significantly increased GST activity, suggesting that GST participated in the protective effect against alcohol-induced liver oxidative injury.

Acetaldehyde is a reactive compound that can interact with the thiol and amino groups of DNA, protein, and lipids. Acetaldehyde adducts, such as 4-HNE, may cause the inhibition of protein function, cause an immune response, inhibit ALDH2 and GST activity, and thereby exacerbate ALD [35, 36]. MDA is one of the lipid peroxidation decomposition products and is widely used as a main marker of lipid peroxidation [37]. The TBARS method is a technique used to detect MDA spectrophotometrically through the reaction of MDA with thiobarbituric acid [22]. A previous study showed that ALDH2 contributes to the prevention of ALD by removing acetaldehyde and lipid peroxidation-derived aldehydes, such as MDA and 4-HNE [38, 39]. The iso-α-acid diet decreased TBARS levels in the liver, suggesting that iso-α-acids intake inhibits the accumulation of lipid peroxide in the liver by increasing ALDH and GST activity.

Nrf2 and its specific repressor Keap1 mediate cellular responses to oxidative stress and regulate the transcription of the genes including enzymes involved in detoxification, antioxidation and ethanol-acetaldehyde degradation. Nrf2, once dissociated from Keap1, translocates to the nucleus and facilitates the expression of target genes by binding to antioxidant responsive elements in their promoter regions [11]. Nrf2 regulates the expression of multiple antioxidant and detoxification genes during oxidative stress. Nrf2 is ubiquitinated by Keap1 and is degraded rapidly by the proteasome; thus, the Nrf2 protein level is low in cells at steady state [40]. When cells are exposed to toxins or oxidative stress, they are detected by Keap1 cysteine residues and then modified. Such molecular events reduce the ubiquitination level of Nrf2 and lead to its stabilization and nuclear accumulation [41]. Nrf2 binds to antioxidant response elements in the nucleus and promotes the transcription of antioxidant and detoxification genes, such as the subunits of GST, ALDH, and others [42]. Our in vitro assay showed that iso-α-acids induced the nuclear accumulation of Nrf2 and the elevation of GST and ALDH enzymatic activities. These results strongly suggest that iso-α-acids protected against liver injury by regulating the Nrf2-Keap1 signaling pathway. Further studies such as the observation of Nrf2 nuclear localization in primary hepatocytes and/or in live samples from iso-α-acids fed animals are required to explain the precise mechanisms involved in Nrf2-mediated antioxidant effects of iso-α-acids. Similar observations using nuclear transport inhibitor or proteasome inhibitor treated-cells and Nrf2-null mice would provide further insight.

Several compounds have been reported as Nrf2 activators. Dimethyl fumarate and bardoxolone methyl are clinically used as a major drug for psoriasis and multiple sclerosis and as a candidate drug for chronic kidney disease, respectively. Plant-derived compounds, including epigallocatechin gallate, sulforaphane, resveratrol and carnosic acid, are also known as activators of the Nrf2-Keap1 signaling pathway and are used as clinical drugs as well as functional food factors [43]. These compounds activate Nrf2 by chemical modification of Keap1 cysteine residues [44]. In particular, sulforaphane upregulates the expression of ALDH and GST genes and protects against alcoholic liver disease via the Nrf2-Keap signaling pathway [21, 45–47]. Since the present study is consistent with these reports, iso-α-acids may modify Keap1 cysteine residues and activate the pathway.

In the previous study, iso-α-acids prevented nonalcoholic fatty liver disease in mice at a dose of 0.5% (w/w) in the diet [48]. And intake of iso-α-acids significantly enhanced ALDH activities at doses of 0.5% or more (S2 Table). We selected the dose of iso-α-acids based on these studies. This concentration is equivalent to approximately 300–500 mg/kg body weight. Commercial beer contains hop-derived iso-α-acids at a concentration of approximately 20–40 mg/L. It was shown that the blood concentration of iso-α-acids reached approximately 0.1 ppm at 30 min after the consumption of 600–800 mL of beer in humans [49]. This study showed that 5 ppm iso-α-acids was effective in hepatocytes. Therefore, the contribution of hop-derived iso-α-acids in beer for the prevention of liver injury may be very small.

## Conclusions

In conclusion, we showed that iso-α-acids intake protected against acetaldehyde-induced liver injury. Iso-α-acids upregulated the expression of the GST and ALDH genes, resulting in increase of their overall activity. Moreover, iso-α-acids promoted the nuclear accumulation of Nrf2. We conclude that iso-α-acids enhanced antioxidation and detoxification and protected against ROS and xenobiotic substances in the liver. Iso-α-acids intake from beer may have some impact on liver protection, although the amount of iso-α-acids in beer is very small. However, the extrapolation of animal data to humans has not been clarified yet in this study. Further investigations, such as animal tests of using ethanol-fed mice, ALDH2 knockout mice, biogenetics, and dose assessment as well as human studies, are required to develop hepatoprotective functional foods containing iso-α-acids.

## Supporting information

S1 Table The effect of iso-α-acids intake on body weight and liver weight in mice. (DOCX)

S2 Table Body weights, serum biochemical parameters, and hepatic biochemical parameters for three doses of iso-α-acids (without acetaldehyde treatments). (DOCX)

S1 Fig Principal component analysis of qFARMS-quantified DNA microarray data. The labels on the x- and y-axes represent PC1 and PC2, respectively, with the proportion of variance in parentheses. Circles: control group, squares: iso-α-acids group.
(TIF)

S2 Fig Significantly enriched GO terms found in the upregulated DEGs induced by iso-α-acids intake. DEGs obtained by a DNA microarray analysis were classified into functional categories based on GO biological process terms using a modified Fisher’s exact test with a Benjamini-Hochberg multiple testing correction method (FDR < 0.05). Count: the number of genes involved.
(TIF)

S3 Fig Significantly enriched GO terms found in the downregulated DEGs induced by iso-α-acids intake. DEGs obtained by a DNA microarray analysis were classified into functional categories based on GO biological process terms using a modified Fisher’s exact test with a Benjamini-Hochberg multiple testing correction method (FDR < 0.05). Count: the number of genes involved.
(TIF)

S1 Raw images (PDF)
