# Supplementary material for: Dietary iso-α-acids prevent acetaldehyde-induced liver injury through Nrf2-mediated gene expression
Source: PLoS One. 2021 Feb 5;16(2):e0246327. doi: 10.1371/journal.pone.0246327 (PMC7864453; doi:10.1371/journal.pone.0246327)
Supplement: S1 Table — (DOCX) [file pone.0246327.s001.docx]

**S1 Table. The effect of iso-α-acids intake on body weight and liver weight in mice.**

|  | **Control** | | |  | **Iso-α-acids** | | |
| --- | --- | --- | --- | --- | --- | --- | --- |
|  | **Mean** | **±** | **S.E.M** |  | **Mean** | **±** | **S.E.M** |
| **Body weight (g)** | **23.41** | **±** | **0.42** |  | **23.13** | **±** | **0.34** |
| **Liver weight (g)** | **0.78** | **±** | **0.03** |  | **0.75** | **±** | **0.04** |

All parameters are shown as the means ± S.E.M (*n* = 5). **P* < 0.05 (vs. the control group)
