# Supplementary material for: Dietary iso-α-acids prevent acetaldehyde-induced liver injury through Nrf2-mediated gene expression
Source: PLoS One. 2021 Feb 5;16(2):e0246327. doi: 10.1371/journal.pone.0246327 (PMC7864453; doi:10.1371/journal.pone.0246327)
Supplement: S2 Table — (DOCX) [file pone.0246327.s002.docx]

**S2 Table. Body weights, serum biochemical parameters, and hepatic biochemical parameters for three doses of iso-α-acids (without acetaldehyde treatments).**

The nine-week-old male C57BL/6NCrSlc consumed each iso-α-acids diet for a week. For serum AST and ALT assay and liver tissue collection, tail vein blood samples and liver samples were collected under deep anesthesia without acetaldehyde treatment. All parameters are shown as the means ± S.E.M (*n* = 3). Different lowercase letters represent significant differences (*P* < 0.05) by one-way ANOVA with the Tukey multiple comparison test.
