# Supplementary material for: Dietary iso-α-acids prevent acetaldehyde-induced liver injury through Nrf2-mediated gene expression
Source: PLoS One. 2021 Feb 5;16(2):e0246327. doi: 10.1371/journal.pone.0246327 (PMC7864453; doi:10.1371/journal.pone.0246327)

Fig. 5A, raw image

Lane 1 and 15 ; Pre-stained Protein Markers (Nakarai)

Lane 2 ; WESTERN-VIEW Western Protein Size Marker (Wako)

Lane 3, 7, and 11 ; Control

Lane 4, 8, and 12 ; Iso-α-acids 5 ppm

Lane 5, 9, and 13 ; Iso-α-acids 25 ppm

Lane 6, 10, and 14 ; Iso-α-acids 100 ppm

anti-Nrf2 blot for cytoplasmic protein

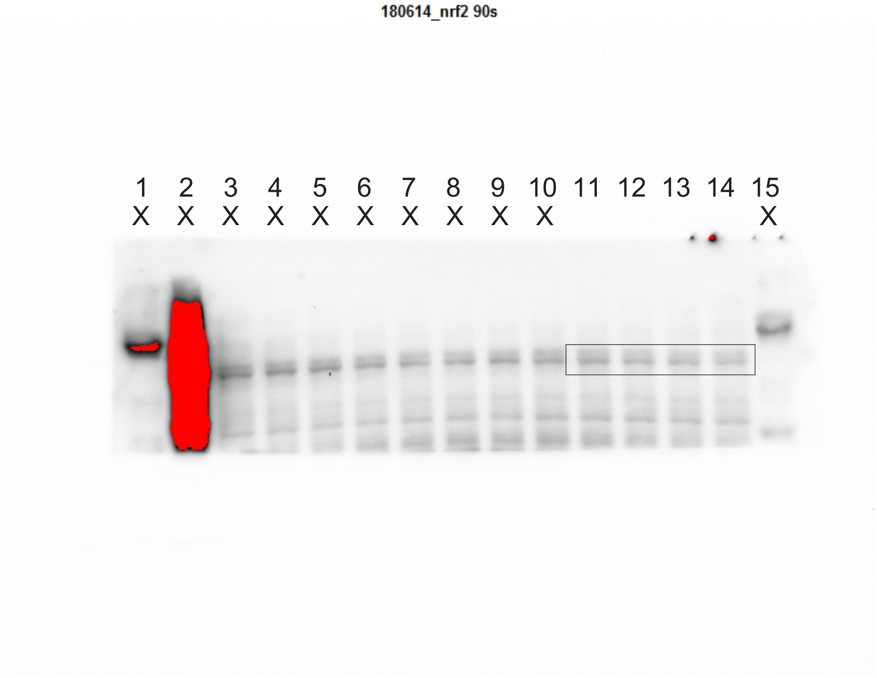

anti-β-actin blot for cytoplasmic protein

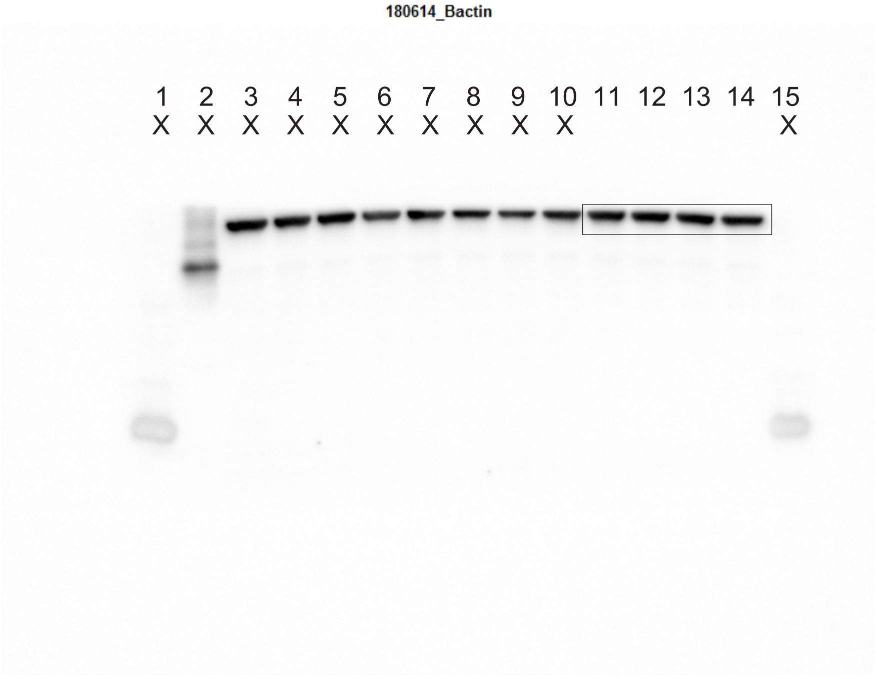

Fig. 5B, raw image

Lane 1 and 15 ; Pre-stained Protein Markers (Nakarai)

Lane 2 ; WESTERN-VIEW Western Protein Size Marker (Wako)

Lane 3, 7, and 11 ; Control

Lane 4, 8, and 12 ; Iso-α-acids 5 ppm

Lane 5, 9, and 13 ; Iso-α-acids 25 ppm

Lane 6, 10, and 14 ; Iso-α-acids 100 ppm

anti-Nrf2 blot for nuclear protein

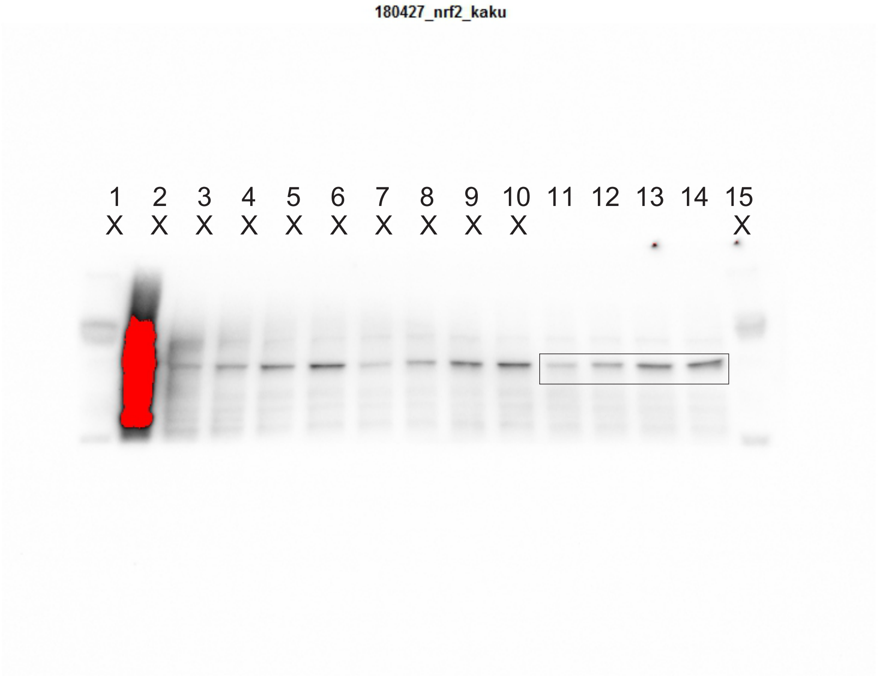

anti-β-actin blot for nuclear protein

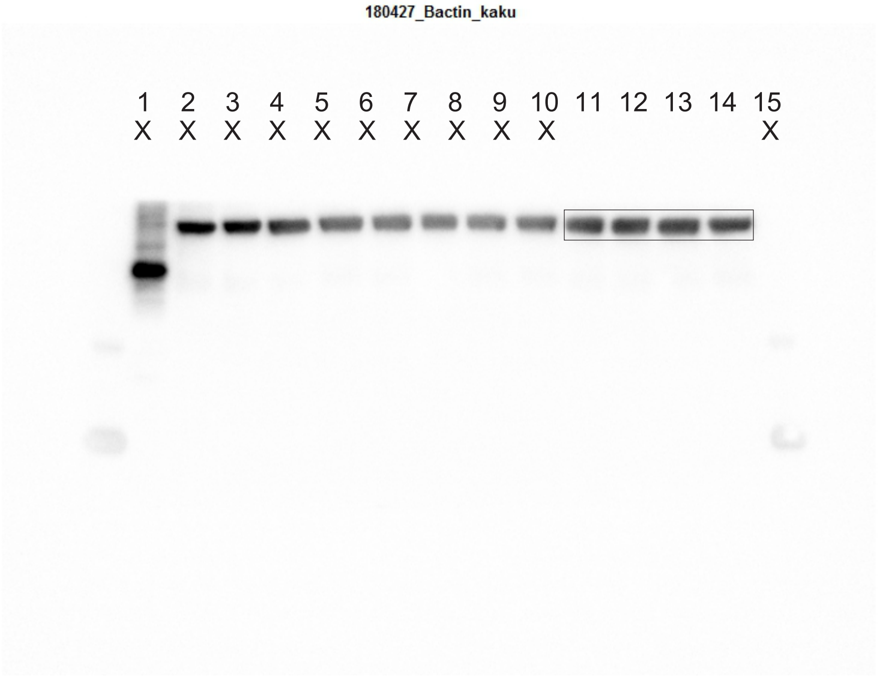

Supplement: S1 Raw images — (PDF) [file pone.0246327.s006.pdf]
